# The flip side of sirtuins: the emerging roles of protein acetyltransferases in aging

**DOI:** 10.18632/aging.102949

**Published:** 2020-03-13

**Authors:** Prabakaran Nagarajan, Mark R. Parthun

**Affiliations:** 1 Department of Biological Chemistry and Pharmacology, The Ohio State University, Columbus, OH 43210, USA

**Keywords:** acetyltransferase, acetylation, aging, Sirtuin, KAT

## Abstract

Protein N-ε-lysine acetylation is is an important post-translational modification that plays critical roles in the regulation of many cellular processes. A role for this modification in the process of aging goes back two decades to the discovery that the yeast NAD^+^-dependent histone deacetylase Sir2 regulates lifespan in yeast. While the Sirtuin family of protein deacetylases has been intensively studied in many model systems and is definitively linked to aging, the enzymes responsible for protein acetylation, protein acetyltransferases (KATs), have not received a similar level of attention. However, a series of recent studies have directly explored the role of specific KATs in aging. These studies have shown that modulation of KAT activity can influence cellular pathways important for aging and directly effect organismal lifespan.

The level of acetylation on a given protein is the result of a balance in the activity of opposing families of enzymes, protein lysine acetyltransferases that attach the acetyl moieties and protein deacetylases that remove the acetyl groups. The idea that protein acetylation plays an important role in the regulation of aging began with the pioneering work on the Sirtuin family of NAD^+^-dependent protein deacetylases. Levels of the yeast histone deacetylase Sir2 correlated with lifespan as increased levels of Sir2 increasing lifespan and deletion of Sir2 decreasing lifespan in *S. cerevisiae* [1,2]. Subsequent studies in model organisms such as, flies, worms and mice, showed that genetic or pharmacological modulation of Sirtuin activity influenced lifespan [3–9]. While a role for protein deacetylases in aging is firmly established, the enzymes on the other side of the equation, the protein lysine acetyltransferases, have not received a proportionate share of research into understanding their potential roles in the regulation of aging.

Protein N-ε-lysine acetyltransferases (KATs) are a diverse family of enzymes [10]. While many of these enzymes were originally identified as histone acetyltransferases, it is now clear that most, if not all, have multiple substrates. From a broad perspective, it is not surprising that KATs are likely to play key roles in the aging process. KATs modify proteins involved in many cellular processes including those linked to the hallmarks of aging [11]. A number of recent studies have directly examined specific KATs for a link to aging.

## Hat1

Hat1 was the first KAT identified (also known as Kat1). It was originally isolated based on its role in the evolutionarily conserved diacetylation of newly synthesized histone H4 during the process of chromatin assembly [12,13]. While Hat1 is essential for viability in mice, a link between Hat1 and aging was identified by the analysis of Hat1^+/-^ heterozygotes [14]. Hat1^+/-^ animals are largely normal at birth but develop a number of phenotypes suggestive of early nset aging within their first year. These phenotypes include lordokyphosis, hind limb paralysis, muscle atrophy, loss of subcutaneous fat and tumor development. Strikingly, Hat1^+/-^ mice have a significantly shortened lifespan of approximately 69 weeks compared to greater than 120 weeks for wild type animals. A direct role for Hat1 in the normal aging process is suggested by the observation that Hat1 expression, at both the mRNA and protein levels, decreases dramatically with age in wild type animals [15]. Although they have opposite effects on protein acetylation, it is intriguing that decreases in Hat1 activity have a similar effect on aging as decreases in Sirtuin activity. This is consistent with observations in yeast where deletions of Hat1 and Sir2 both lead to loss of telomeric silent chromatin structure [16]. The mechanism(s) by which Hat1 influences aging are not clear as Hat1 is involved in multiple cellular process important to aging at the cellular level. These include transcriptional regulation, DNA damage repair, genome stability and mitochondrial function [14,15,17–21]. In addition, a recent proteomic analysis indicates that Hat1 influences the acetylation state of a number of proteins known to be important for mammalian aging (Agudelo Garcia,et al, bioRxiv doi: https://doi.org/10.1101/825539).

## CBP/p300

The paralogs CBP and p300 are transcriptional coactivators that possess protein acetyltransferase activity. CBP and p300 participate in multiple signaling pathways and are key factors in disease states such as cancer and neurodegeneration [22]. Several lines of evidence indicate that these KATs are also critical factors in several aspects of aging. First, p300 has been shown to be an important regulator of cellular senescence, which is an important driver of decreased tissue function during aging [23–25]. Second, acetylation of several proteins by p300 and/or CBP have been shown to be involved to be involved in aging-related processes, including WRN, C/EBPα and TAU [26–29]. Third, lifespan extension in model organisms, through either dietary/caloric restriction or pharmacological mimetics of dietary restriction, requires CBP and p300 [30–36]. Finally, studies in *C. elegans* have directly demonstrated that reduced expression of CBP or p300 shortens lifespan [37–40].

## CLOCK

The KAT protein CLOCK is an integral component of the of the molecular clock that maintains circadian rhythms [41]. Circadian rhythms play an important role in a variety of processes, including stress responses, immune function, metabolism and sleep regulation. Disruptions of the circadian rhythms can have serious pathological consequences including improper metabolism, sleep disorders, cardiovascular disease and neurodegenerative diseases [42–44]. Mutational analyses in flies and mice have indicated that loss of CLOCK activity is linked to age-dependent tissue defects. In flies, CLOCK is required in pacemaker neurons to prevent premature locomotor aging. Interestingly, this effect is independent of the role of CLOCK in the circadian rhythm [45]. In mice, expression of a CLOCK mutant lacking exon 19 (Clock^Δ19/Δ19^) results in accelerated aging in both the heart and liver [46,47].

## Chameau

Chameau (Chm) is the *D. melanogaster* homolog of Hbo1 (KAT7). Hbo1 is a MYST family acetyltransferase that functions in regulating gene expression and DNA replication [48]. In a recent study examining changes in metabolism and histone acetylation during aging, it was found that flies with a catalytically inactive Chm mutation had a significant increase in lifespan. It was proposed that the Chm mutation extended lifespan through the attenuation of transcriptional changes associated with aging [49].

These recent studies have now shown that several KATs are directly linked to the aging process and that genetic and pharmacological manipulation of KATs can influence lifespan. Our understanding of the link between KATs and aging clearly has a long way to go to match our understanding of Sirtuins. Important questions that need to be addressed include determining the relevant aging-related cellular processes that each KAT functions in and identifying aging-relevant substrates for each KAT. It will take intensive investigation to decipher the molecular mechanisms underlying the influence of KATs on aging and lifespan.
